# Retraction: Circular RNA hsa_circ_0013958 functions as an oncogenic gene through modulating miR-532-3p/WEE1 axis in hepatocellular carcinoma

**DOI:** 10.3389/fonc.2024.1477868

**Published:** 2024-09-05

**Authors:** Frontiers Editorial Office

The journal retracts the 15 April 2021 article cited above.

Following publication, concerns were raised regarding the validity of the data in the article. The authors failed to provide the raw data and a satisfactory explanation during the investigation, which was conducted in accordance with Frontiers’ policies. Given the concerns about the validity of the data, and the lack of raw data, the editors no longer have confidence in the findings presented in the article. This retraction was approved by the Chief Executive Editor of Frontiers.

The authors did not agree with the retraction.

